# Reconstructing the expression of placement events in Danish as a second language

**DOI:** 10.3389/fpsyg.2022.922682

**Published:** 2023-01-09

**Authors:** Teresa Cadierno, Iraide Ibarretxe-Antuñano, Alberto Hijazo-Gascón

**Affiliations:** ^1^Department of Language and Communication, Center for Language Learning, University of Southern Denmark, Odense, Denmark; ^2^Departmento de Lingüística y Literaturas Hispánicas, Instituto de Patrimonio y Humanidades, University of Zaragoza, Zaragoza, Spain; ^3^School of Politics, Philosophy, Language and Communication Studies, University of East Anglia, Norwich, United Kingdom

**Keywords:** placement events, second language acquisition, Danish, Spanish, caused-motion, cross-linguistic influence, construction grammar

## Abstract

Cross-linguistic research on event typology has revealed considerable variation in the linguistic conceptualization of placement events. Previous studies on second language acquisition have primarily dealt with the semantic re-categorization of placement verbs in a second language, but placement constructions have received less attention. The present study fills this gap by examining the constructions used by Spanish learners of L2 Danish (B1 and B2 levels) and by monolingual speakers of both languages. Data were elicited by means of the PUT task consisting of oral video descriptions and then classified into six main placement construction categories based on their frequency and structure. Results from the learner group suggest learning difficulties when reconstructing the expression of placement events in L2 Danish. In contrast to L1 Danish data, learners (i) kept using their L1 Spanish basic placement construction more often, (ii) avoided semantically more complex constructions, (iii) employed fewer spatial particles, (iv) showed difficulties in selecting the L2 appropriate spatial particles for specific placement scenes, and (v) used non-caused motion constructions. These findings suggest the creation of a linguistic conceptualization pattern on the part of the learners that is different from the respective L1 and L2 monolingual patterns, thus providing further empirical support for proposals arguing that bilinguals’ multicompetence is not equivalent to those of two monolinguals.

## Introduction

In the last decade, cross-linguistic research on event typology has turned its attention to the study of placement events, a type of caused-motion event where an agent causes an object to move to a specific location, as in *John puts a cup on the table*. The investigation of placement events is an interesting area for the study of second language acquisition (SLA), as first language (L1) research (e.g., Kopecka and Narasimhan, 2012) has revealed considerable variation in the linguistic conceptualization of this domain by native speakers (NSs) of different languages. For example, in Spanish it is common to use general caused-motion verbs such as *dejar* ‘leave (on a place)’ or *poner* ‘put’ (Ibarretxe-Antuñano, 2012) whereas in Danish, posture verbs like *lægge* ‘put horizontally’ or *stille* ‘put vertically’ are commonly used (Cadierno et al., 2016).

Previous SLA research on the expression of placement events by second language (L2) learners has primarily dealt with the semantic re-categorization of placement verbs (e.g., Gullberg, 2009a; Cadierno et al., 2016; Ibarretxe-Antuñano et al., 2016; Lewandowski and Özçalışkan, 2021). Less attention has been given to the study of the linguistic constructions that learners use when talking about placement. In a previous study, Hijazo-Gascón et al. (2016) examined the types of constructions that Danish L1 learners of L2 Spanish used when describing placement events and compared them to the constructions used by L1 Danish and L1 Spanish NSs. The present study builds on this research by examining the types of constructions that Spanish L1 learners of L2 Danish employ when describing the same placement events.

## Background

### The caused-motion construction

Caused-motion has been extensively studied from Talmy’s (1985, 1991) cognitive semantics perspective. It involves an agent that causes an object to move to a specific location in space. Some basic semantic components have been identified as part of these events (Talmy, 1985; Jackendoff, 1990; Narasimhan et al., 2012). For example, the Figure is the object that is caused to move; the Agent is the entity that causes the change of location of the object; the Ground is the location with respect to which the Figure moves; Causation is what triggers the placement; Motion is the act of moving itself; and the Path is the trajectory followed by the Figure.

Placement events are a specific type of caused-motion events and the focus of the present study. Previous research has identified cross-linguistic variation in the encoding of these events. Choi and Bowerman (1991) examined Korean and English lexicalization patterns, i.e., systematic ways of expressing and encoding semantic components by means of the resources available in each language. They found that English speakers focused on the characteristics of the Ground, whether it is two-or three-dimensional. For example, *She puts the cup on the table* indicates a support relationship between the Figure and the Ground, while *She puts the cup in the cupboard* encodes a container relationship. By contrast, what is relevant for Korean speakers is whether the relation between the Figure and the Ground is tight-fit or loose-fit. Accordingly, they frequently encode placement events by using the verb *kkita* ‘put tightly’ or *nehta* ‘put loosely’.

Further research on the lexicalization of placement events shows that the differences are not exclusive to this language pair and that the encoding of this type of event varies across languages. The studies collected in Kopecka and Narasimhan (2012) reveal differences in the lexical semantics of placement verbs and their degrees of specificity. For example, Swedish has posture verbs that express the orientation of the Figure with respect to the Ground, e.g., *sätta* ‘set’, *ställa* ‘stand’, and *lägga* ‘lay’ (Gullberg and Burenhult, 2012). Other languages like English have both a general verb –*put*– that is more widely used and a set of placement verbs –*set*, *lay*, *stand*– generally used for contrastive and pragmatically motivated reasons (Gullberg, 2011a).

Placement events are also an interesting area of research from a multimodality perspective. In a study with Dutch and French speakers, Gullberg (2011a) found that the gestures of Dutch-speakers focused on the Figure being placed and its orientation while French speakers’ gestures focused on the Path of the movement. In a study comparing the verbal and gestural expression of self-motion –when the event involves a Figure that moves voluntarily (i.e., *The girl runs*) – and caused-motion –when the Figure is moved by an Agent (i.e., *The boy pushed the girl*) in German and Spanish, Lewandowski and Özçalışkan (2018) found that caused-motion favored the conflation (i.e., co-occurrence) of several semantic components in gesture in both languages. The authors attributed this to the relevance of force dynamics, i.e., how the force is applied to a Figure to start the movement (Talmy, 1988). As noted by Ibarretxe-Antuñano (2012), force dynamics seems to be a particularly relevant semantic component in Spanish, where several fine-grained distinctions are made, particularly in contrast to other components such as Manner of motion.

Cross-linguistic differences in the expression of placement events are not limited to verb choice. Distributed semantics of spatial information (Sinha and Kuteva, 1995) can also apply to these events. An insight into the whole construction is necessary to achieve a complete overview of how languages encode placement. Verbs with a high degree of semantic specificity are very informative about the relation between the Figure and the Ground and, therefore, leave little room for variation in the preposition. On the other hand, general verbs tend to be combined with prepositions that specify the Figure-Ground relation. Hickmann and Hendriks (2006), for example, have argued that it is rare for both the verb and the preposition to be general. This, however, does not seem true in some languages. In Spanish the use of general verbs such as *poner* ‘put’ and *dejar* ‘leave’ is frequent in combination with general prepositions such as *en* ‘location (in, on, at)’ (Ibarretxe-Antuñano et al., 2016).

Following this line of research, the present study focuses on the placement construction on the whole, i.e., on all the encoding elements that conform to the placement structure. The term “construction” is used in the sense of Goldberg’s (1995, 2006) Construction Grammar. In this vein, we investigate the stored (clause-level) form and meaning patterns that are frequently used by speakers to describe placement events as illustrated in (1).

**Table:** 

(1)	*Mary pushed the ball out of the park*		
	Form	Meaning	Construction label
	Subj V Obj Obl_path/loc_	X causes Y to move Z_path/loc_	Caused-motion

### How are placement events acquired?

#### L1 acquisition

There are different factors in shaping the semantic development of placement events in child language acquisition. In a study with French-speaking children (aged 3–5) and adults, Hickmann and Hendriks (2006) and Hickmann (2007) found that French children overgeneralized neutral verbs (e.g., *mettre* ‘put’) and used fewer specific verbs than French adults, but the frequency of use of these verbs increased with age. These specific verbs used by adults mainly encoded Manner (e.g., *accrocher* ‘hook’, *emboîter* ‘fit’). Interestingly, Gullberg et al. (2008) showed that French children and adults gestured similarly. They gestured with Path alone information when Manner and Path were equally important in the motion event, especially in downward motion, and with Manner alone information for Manner salient events and crossing events. Children included in some cases more Manner information (in Path and Manner conflated gestures), but this tendency decreased with age.

In Dutch, Gullberg and Narasimhan (2010) found that children (aged 3–5) overused the verb *leggen* ‘lay’ and underused the verb *zetten* ‘set/stand’, and that children and adults gestured differently. Children’s gesture patterns mirrored their verb use, i.e., when overusing the general verb they gestured about Path and there were lesser object-incorporating gestures. The developmental pattern found shows an evolution from a single semantic component system –caused-motion– to a two semantic component system –caused-motion and object information– used by the adults.

In a cross-linguistic study with children speaking eight different languages, Slobin et al. (2011) found an interaction between the ease of learning language categories and how each language encodes this information. For example, both Tzeltal and German encode information about the Figure orientation in the verb, but German-speaking children showed more difficulties in the distinction of this type of verb than Tzeltal-speaking children. It seems that the structure of Tzeltal, as a verb-framed language, encourages its speakers to attend to verbs at an early age, while German-speaking children are not as oriented to verbs and need more time to make the semantic distinctions between *legen* ‘lay’ and *stellen* ‘make stand’. In the case of a different verb-framed language, Hernández Sánchez and Ibarretxe-Antuñano (2017) found that 3, 4, and 5 year-old Spanish speaking children acquired the constructions and semantic distinctions adult Spanish speakers employ for placement events (e.g., *dejar en* ‘leave on’, *meter en* ‘put in’, and *tirar a* ‘throw to’).

Other factors that play a role in the L1 acquisition of placement events are input frequency and semantic transparency. Narasimhan and Gullberg (2011) compared Dutch and Tamil placement verbs in groups of children of 2 and 5 years old and adults. Both languages present general and specific posture verbs for placement but their morphosyntactic characteristics and frequency usage differ. Dutch verbs are generally semantically less transparent, i.e., it is not easy to infer their meaning components, and tend to be formed by one morpheme (e.g., *leggen* ‘lay’). Speakers prefer specific placement verbs and use them very frequently, e.g., when describing how a bottle is put, they will specify the orientation of the object with *leggen* ‘lay’ or *zetten* ‘stand’. Tamil verbs, on the other hand, are more transparent, since they are formed by two morphemes: one encodes the Cause and the other, the Result (e.g., *nikka veyyii* ‘make stand’). Tamil speakers tended to use general verbs, e.g., *veyyii* ‘put’ to describe how a bottle is put, while specific caused-motion verbs encoding the posture were reserved for contrasts or non-canonical positions. In Narasimhan and Gullberg’s (2011) study, children’s data revealed that Dutch-speaking children overused the posture verb *leggen* ‘lay’ to cover all vertically and horizontally placement events whereas Tamil-speaking children employed infrequent specific posture verbs appropriately. Narasimhan and Gullberg (2011) concluded that semantic transparency was more relevant than input frequency in the expression of placement events.

#### L2 acquisition

The acquisition of placement events in an L2 has been tackled from different angles^1^. Earlier studies on the L2 acquisition of placement events examined how speakers whose L1 presents a more general system for placement acquire an L2 with a more specific system, i.e., a system with elements that encode more specific semantic components, e.g., Swedish. The specificities of the L2 are expected to be particularly challenging, in agreement with Stockwell et al. (1965), who predicted greater acquisition difficulty of split forms –one item in the L1 becomes two in the L2– rather than coalesced forms –two items in the L1 become one in the L2. For instance, Viberg (1993) found that learners of L2 Swedish tended to replace the whole set of placement verbs –with similar distinctions to those of Dutch and German– with one of these posture verbs. The semantic complexity of the learners’ L1, i.e., the extent to which the L1 contains one general placement verb (a more simple system) or two or more caused posture verbs (a more complex system), emerged as an important factor in their lexical choices. Participants whose L1 had a more complex system, i.e., a system with two verbs that roughly correspond to the Swedish verbs *lägga* ‘lay’ and *ställa* ‘stand’ (L1 Polish) used L2 Swedish verbs more appropriately than participants whose L1s lacked posture-related distinctions (L1 Finnish and Spanish). In a study on the acquisition of L2 Dutch placement events by adult English speakers, Gullberg (2009a) found that the existence of specific cognate verbs did not facilitate the acquisition of L2 Dutch posture verbs. Learners still used constructions with the verb *doen* ‘do’ and other non-placement verbs, and overgeneralized the placement verb *zetten* ‘set’ over *leggen* ‘lay’. As far as gesture is concerned, English participants kept their native patterns, i.e., Path towards Goal gestures, instead of encoding the Dutch patterns, i.e., posture of the Figure. Gullberg (2009b, 2011b) focused on the development from a more specific system to a more general one. German and Dutch learners of L2 French produced target-like use of placement verbs. However, the influence of the L1 appeared in the gesture patterns, which were in the process of restructuring, i.e., in the process of undergoing a conceptual change (Pavlenko, 2011). These results show that some semantic restructuring is also needed when the learners move from a more specific system to a more general one.

Semantic restructuring is also one of the topics Ji and Hohenstein (2014) discussed in relation to the acquisition of motion events at different levels of proficiency in L2 Chinese by L1 English speakers. Their results revealed a moderate influence of the L1 (English) on the L2 (Chinese). However, the L2 speakers did not rely completely on the L1 construction but also employed other types of constructions, some of which were atypical in Chinese. In fact, learners, even those who were advanced, hardly ever used one of the two most prototypical constructions for caused-motion in Chinese; i.e., the most complex construction was avoided.

Lemmens and Perrez’s (2018) study on locative events constructions in L2 Dutch added further support to previous findings. Dutch learners at lower levels of proficiency preferred, and even overused, the presentational construction with *er zijn* ‘there is/are’. With respect to the encoding of locative information in complex sentences, L1 Dutch speakers tended to chain individual locative events whereas L1 French speakers talked about one entity and gave information about that entity. Results revealed that learners became progressively more aware of the target language patterns for locative events with regard to lexis, constructions and information structure, and that they were in between the two main tendencies found in NS of Dutch and French, a developmental pattern also documented in other studies (e.g., Treffers-Daller and Tidball, 2016; Madlener-Charpentier, 2022).

The restructuring tendencies discussed so far showed that learners have difficulties in shifting from a more general to a more specific placement system (e.g., Spanish to Danish). However, Cadierno et al. (2016) also found that moving from a more specific to a more general system (e.g., Danish to Spanish) was equally challenging. In this line of research, Hijazo-Gascón et al. (2016) extended the study of placement events beyond the verb and examined the whole range of placement constructions employed by Spanish and Danish NS and by Danish learners of L2 Spanish. They found that the distribution of different types of constructions varied across the two languages. The construction [Sub V Obj Obl_loc_] as in *El hombre pone el libro en la mesa* ‘The man puts the book on the table’ was clearly the most frequent choice in Spanish NS data. In contrast, Danish NS data revealed preference for two different types of constructions. One type coincided with the preferences in Spanish; that is, the construction [Sub V Obj Obl_loc_] as in *Hun sætter kopen på bordet* ‘She (vertically) sets the cup on the table’. The other type showed the semantically richer construction [Sub V Obj Obj_path_ Obl_loc_] as in *Hun sætter bogen op* *på reolen* ‘She (vertically) sets the book up on the shelf’. The latter includes an additional element, Obl_path_. Based on these results, Hijazo-Gascón et al. (2016) adopted the term “Basic Placement Constructions (BPC)” to refer to those constructions (i.e., pairings between form and meaning) that are more frequently used for the encoding of a placement event in a language. Regarding the L2 data, the study showed that learners: (i) overgeneralized the use of the general verb *poner* ‘put’ for all scenes, (ii) misused or were not aware of the semantic categorization preferences in Spanish NS for support (*dejar* ‘leave’), for containment (*meter* ‘put in’), and intentionality (*caerse* ‘fall’ + clitic for accidentally dropping vs. *dejar caer* ‘let fall’ vs. *tirar* ‘throw’ for deliberately dropping), and (iii) employed locative particles more often (e.g., *dentro* ‘inside’, *encima* ‘on top of’) and widely (*abajo* ‘down’, *arriba* ‘up’). Hijazo-Gascón et al. (2016) concluded that Danish learners have successfully acquired the BLC in Spanish but have not yet grasped the whole rhetorical style in Spanish (sensitive to support, containment, and, above all, intentionality; see Ariño-Bizarro and Ibarretxe-Antuñano, 2020, under review).

In short, previous L2 research generally shows that the process of reconstructing a second language is a complex one because, during development, L2 verb meanings and L2 constructions are in direct competition with the learners’ L1. The process of reconstructing a second language involves learning a new set of conventionalized form-meaning mappings, i.e., learning the specific linguistic means employed by the NS of the target-language to construe given situations and events (Ellis and Cadierno, 2009). More specifically, L2 learners need to learn (a) the specific meanings of the L2 placement verbs and detect possible differences between the L1 and L2 semantic distinctions, and (b) the specific syntax-semantic mappings of L2 constructions and again detect possible cross-linguistic differences in the L1 and L2 mappings.

The current investigation builds on Hijazo-Gascón et al. (2016) study by examining how Spanish learners of L2 Danish deal with placement constructions. The main objective is to investigate whether the expression of placement events at the construction level is challenging in both directions of acquisition, i.e., when the L2 presents a more or less complex semantic system of placement events.

## Aim and research questions

The present study contributes to L2 cross-linguistic research on the expression of placement events by examining the constructions that Spanish learners of L2 Danish use when describing placement events and comparing them to the constructions used by L1 Spanish and L1 Danish NSs. Thus, this study departs from the more traditional verb-centered approach and examines the types of constructions that learners use to encode placement events. Our study thus follows the path initiated by previous work that has adopted a more constructional approach to the study of spatial language (e.g., Cadierno, 2010; Ji et al., 2011; Hijazo-Gascón et al., 2016; Lemmens and Perrez, 2018). The study addressed the following research questions:

RQ 1. What types of constructions were used by the three participant groups (L1 and L2 Danish, L1 Spanish) when describing placement events?
RQ 2. Were there differences in the frequency with which these three participant groups used the different types of constructions? More specifically,
RQ 2.a. Were there differences in the relative frequency with which *each* participant group used the different types of constructions?
RQ 2.b. Were there differences in the relative frequency with which each type of construction was used *across* the three participant groups?

## Materials and methods

### Participants

Thirty-eight speakers participated in this study: 14 NSs of Danish, 10 NSs of Spanish and 14 adult Spanish learners of L2 Danish. Danish and Spanish NSs were university students at the University of Southern Denmark and the University of Zaragoza (Spain). These participants can be characterized as functional monolinguals as they were not studying English or any other L2 at the time of data collection and they only used Danish and Spanish, respectively, in their daily lives (*cf*. Brown and Gullberg, 2012). Danish NSs (12 women, 2 men), aged between 19 and 26, had no prior knowledge of Spanish. Spanish NSs (8 women, 2 men), aged between 18 and 44, had no prior knowledge of Danish. The L2 Danish learner group consisted of speakers who were studying Danish as a foreign language at the *Escuela Oficial de Idiomas* (Official School of Languages, a national organic law regulated institution for teaching foreign languages in Spain) in Madrid. They were 8 women and 4 men, with ages between 22 and 46 and university backgrounds. Their level of L2 proficiency in Danish was between CEFR B1 and B2 levels. As shown by a language background questionnaire, 12 of these learners had lived and studied Danish in Denmark for a period ranging from 1 month to 4 years, mainly for tourism and study in the short stays and for university exchanges (Erasmus Program) and work for those who spent longer periods (4 participants lived in Denmark for longer than 1 year). Eight of them used Danish out of the classroom context (with friends, partners, in-laws, or in work emails). All learners but one had advanced knowledge of English and 8 reported some knowledge of other languages such as German, French, and Italian, at different levels. However, all of them reported that Spanish was their first language and the language used with their parents, family, friends (four of them also English, and one of them French and Italian), and work colleagues (except one who used also German and five also English).

### Procedure

Data for the present study consisted of individually videotaped oral descriptions of 31 short video clips. These clips are part of the “PUT project” stimuli tool, a set of 63 (plus three warming-up) clips showing placement and removal events developed at the Max Planck Institute for Psycholinguistics in Nijmegen (Bowerman et al., 2004; Kopecka and Narasimhan, 2012). In our subset of placement events, each video shows a human actor performing a caused-motion event such as put cup on table [001], pour liquid into container [020] and put stone into pocket [016] (see Appendix 1 for the full list of caused-motion events and [codes]). The scenes varied along a series of dimensions such as the nature and spatial configuration of the Figure and the Ground and the Manner in which the Figure was moved. The videos were presented in three randomized orders to each group of participants to avoid any possible elicitation bias in participants’ responses. Each participant saw one video clip at a time on a HP Envy17 computer screen and described it to the experimenter. Answers were videotaped with a Sony Handycam HDR-PJ30VE. The learners were told that if they did not know the name of a given object in the video, they could use words like “that” or “that thing” or could ask the experimenter. If asked, the experimenter provided the Danish nouns for the Figure object or the Ground (e.g., Danish *bord* “table”) but never for the L2 verbs required to describe the placement event in question.

### Data analysis

The participants’ oral descriptions were transcribed and coded based on the MPI guidelines for the PUT project (Bowerman et al., 2004). Additionally, for the purpose of the present study, we coded each oral description according to the type of construction that was used. On the basis of the oral descriptions provided by the three participant groups (i.e., the two NS groups and the L2 Danish learner group), six main types of constructions were identified (see “Types of placement constructions used by the three participant groups”). Multinomial logistic regression models with participant group as independent variable and type of construction as dependent variable were used to examine possible group differences in terms of frequency of type of construction. This statistical model was chosen given that the dependent variable consisted of more than two categorical outcomes. Finally, the frequency of spatial particles used by the three participant groups was analyzed.

## Results

### Types of placement constructions used by the three participant groups

Table 1 shows the six main types of placement constructions (PC) that were identified on the basis of the descriptions provided by the participant groups. These six types of constructions share the basic placement construction meaning, “X causes Y to move Z_path/loc_.” This basic meaning may be then modified (either reduced or enriched) in some of these PCs by means of the particular elements included in each type. For example, the inclusion of a Prepositional Phrase may further specify the instrument used by the agent (X) to change the object’s (Y) position. Each PC type provides a structural description of the construction and an illustrative example taken from the elicited data.

**Table 1:** Types of linguistic placement constructions (PC) identified in the data.

PC types	Description	Examples	
		Spanish	Danish
PC #1	[NP/PRO + V + NP/PRO (DO) (+ PP/Gerund (INST))]	*El hombre se pone el sombrero* the man clitic.3sg puts the hat ‘The man puts on the hat’	*En mand kaster en bog* a man throws a book ‘A man throws a book’
PC #2	[NP/PRO + V + NP/PRO (DO) + PP (WHERE) (+PP/gerund (INST)]	*El hombre pone el libro en el suelo (con la mano)* the man puts the book loc the floor with the hand ‘The man puts the book on the floor (with the hand)’	*Hun sætter kopen på bordet (med en grilltang)* she puts.horizontally cup.the on table.the (with a grilltong) ‘She sets the cup on the table (with grill tongs)’
PC #3	[NP/PRO + V + NP/PRO (DO) + PART (PATH) + PP (WHERE) (+ PP/Gerund (INST))]	*El hombre pone el libro encima de la mesa (con la mano)* the man puts the book on.top of the table with the hand ‘The man puts the book on top of the table (with his hand)’	*Hun sætter bogen op på reolen* she puts.vertically book.the up on shelf.the ‘She (vertically) sets the book up on the shelf’
PC #4	[NP/PRO + V + NP/PRO (DO) + PART (+ PP/gerund (INST))]	–	*Hun tager huen på* she takes hat.the on ‘She puts the hat on’
PC #5	More complex constructions involving more than one PP/particles	–	*Hun sætter et krus fra sig* *på bordet* she puts.vertically a mug from herself on table.the ‘She (vertically) sets a mug away from herself on the table’ *Hun sætter koppen tilbage* *hen på bordet* she puts.vertically cup.the back over on table.the ‘She (vertically) sets the cup back over on the table’
PC #6	Other type of non-caused-motion constructions/descriptions	*Una persona toma la copa y el agua está en la mesa* one person takes the cup and the water is.stative loc the table ‘A person takes the cup and the water is on the table’	*En mand bærer nogle bøger men en af dem falder* a man carries some books but one of them falls ‘A man carries some books but one of them falls’

NP = noun phrase; PRO = pronoun; DO = direct object; Inst = instrument; PP = prepositional phrase; PART = particle.

As shown in Table 1, placement construction #1 (PC #1) consists of an NP/PRO that encodes the Agent, a caused-motion verb, and a noun phrase/pronoun or the absence of an explicit overt NP (DO) that encodes the Figure; additionally, this type of construction may contain a prepositional phrase indicating the instrument (e.g., the hand). This type of construction does not incorporate information about the Ground, i.e., the location where the Figure is placed. Placement construction #2 (PC #2) contains the same elements as PC #1 plus a prepositional phrase (PP) that encodes where the Figure was moved to, i.e., the Ground. In our data, this Ground typically encodes the endpoint of the caused-motion (e.g., the table). This construction can also provide information about the Instrument. Placement construction #3 (PC #3) contains the same elements as PC #2 together with a spatial “particle” that encodes the Path of the caused-motion. The term “particle” in this study is coined as a shorthand, neutral, and cover term to include elements with different morpho-syntactic features in Spanish, Danish, or any other language for that matter (e.g., Romance adverbial pronouns derived from Latin IBI and INDE). Elements such as Danish *ned* ‘down’ and *op* ‘up’ (usually referred to in the Talmian framework as satellites; see also, Hansen and Heltoft, 2011) or Spanish locative adverbs (generally treated as problematic in the literature; see Morimoto and Pavón Lucero, 2003) are included under this cover term in order to facilitate this contrastive and intertypological study in L2 acquisition^2^. Placement construction #3 (PC #3) also admits the inclusion of an Instrument (e.g., the tongs). Placement construction #4 (PC #4) contains a NP/PRO, a verb, a NP/PRO (DO) and a spatial particle that encodes the Path of motion (generally, the Goal or the Ground, i.e., the final situation they end up located). This construction is typically used in Danish for the description of dressing events, but it is not possible in Spanish. Placement construction #5 (PC #5) comprises a series of more complex constructions in which there are more elaborate descriptions of the Path of caused-motion by means of spatial particles (e.g., *over* ‘over’, *ned* ‘down’) and/or the Ground (e.g., *fra sig på bordet* ‘(away) from herself on the table’). Finally, a sixth category, placement construction #6 (PC #6), was added to compile the use of any other type of construction which does not encode caused-motion.

In all six types of constructions, the DO can consist of a NP or a pronoun. In Danish, the word order would be the same in both cases, i.e., the DO is always after the verb (e.g., *En mand kaster en bog* ‘A man throws a book’*/En mand kaster den* ‘A man throws it’). In Spanish, on the other hand, the word order is different. If it is a NP, it is placed after the verb whereas if it is a pronoun, it is placed before the verb (e.g., *El hombre se pone el sombrero* ‘The man puts on the hat’ vs. *El hombre se lo pone* ‘The man it puts on’). We have considered both word orders as belonging to the same type of construction as they encode the same semantic content.

Table 2 summarizes the total number of different placement constructions used by the three participant groups. PC #2 (1,200 tokens) was the most widely used type of construction, followed by PC #3 (918 tokens), PC #6 (420 tokens), and PC #4 (316 tokens). PC #1 (85 tokens) and PC #5 (95 tokens) were the least frequently used types of constructions.

**Table 2:** Total number of construction types per participant group.

Language group	PC#1	PC #2	PC #3	PC #4	PC #5	PC #6	Total
L1 Danish	17	382	453	180	80	84	1,196
L1 Spanish	49	406	165	0	0	12	632
L2 Danish	19	412	300	136	15	324	1,206
Total	85	1,200	918	316	95	420	3,034

### Relative frequency of placement construction type per participant group

**Figure 1:**
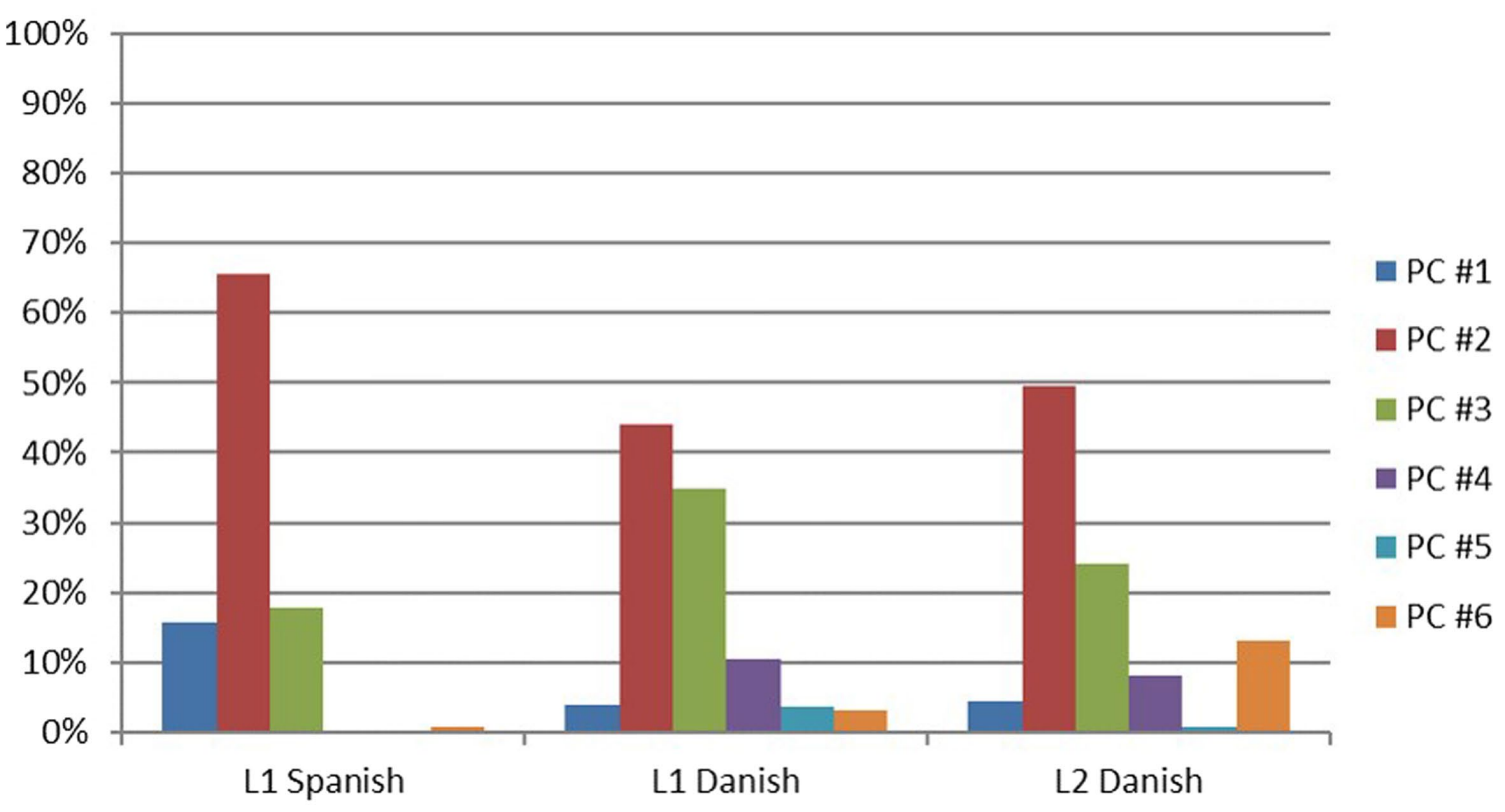
Percentage of placement construction use in the three speaker groups.

Table 3 and Figure 1 show the relative frequency with which each participant group used the different types of placement constructions.

**Table 3:** Relative frequency of type of construction per participant group.

Language group	PC #1	PC #2	PC #3	PC #4	PC #5	PC #6	Total (%)
L1 Danish	1%	32%	38%	15%	7%	7%	100
L1 Spanish	8%	64%	26%	0%	0%	2%	100
L2 Danish	2%	34%	25%	11%	1%	27%	100

With respect to the two NS groups, the L1 Spanish NS group predominantly used only one type of construction as their Basic Placement Construction (BPC): PC #2. In the L1 Danish NS group data, two predominant constructions arise as their BPCs: PC #2, i.e., the same as for Spanish NSs, and PC #3, a more semantically rich construction. Additionally, L1 Danish NSs employed two types of more semantically complex constructions: PC #4 and PC #5. There were not present in L1 Spanish NS data. Finally, L1 Spanish NSs used PC #1 to describe dressing events as in *El hombre se pone el sombrero* ‘The man puts on the hat’ more often than L1 Danish NS. This latter group also used this type of construction to describe ‘dropping’ and ‘throwing’ events such as *En mand kaster en bog* ‘A man throws a book’.

Regarding the L2 Danish group, when comparing their performance to L1 Danish NSs, the results showed that the learners exhibited a slightly more frequent use of PC #2 (i.e., the BPC in L1 Spanish) and a less frequent use of PC #3 (i.e., a rather infrequent placement construction in L1 Spanish). In addition, learners used PC #6, a group of constructions that do not encode the cause of motion, more frequently than the two NS groups. For instance, the example *En mand bærer nogle bøger men en af them falder* ‘A man carries some books and one of them falls’ focuses on the ‘carrying’ and ‘falling’ parts of the event rather than on the ‘placing’ result.

Figure 1 visually summarizes the distribution of these construction types among the three language groups.

### Relative frequency of type of placement construction across participant groups

Table 4 summarizes the relative frequency with which each type of construction was used across the three participant groups.

**Table 4:** Relative frequency of type of construction across participant groups.

Language group	PC #1	PC #2	PC #3	PC #4	PC #5	PC #6
L1 Danish	20%	32%	49%	57%	84%	20%
L1 Spanish	58%	34%	18%	0%	0%	3%
L2 Danish	22%	34%	33%	43%	16%	77%
Total	100%	100%	100%	100%	100%	100%

As shown in Table 4, only one type of construction, PC #2, was consistently used by the three groups with similar frequency (32% for L1 Danish; 34% for L1 Spanish; and 34% for L2 Danish). Examples in (2) illustrate the use of PC #2 for the scene Put box up on shelf [006].

**Table:** 

(2)	a.	L1 Spanish:								
		*Deja*	*la*	*caja*	*de*	*cartón*	*en*	*la*	*estantería*	
		leaves	the	box	of	cardboard	Loc	the	shelf	
		‘(He/she) leaves the cardboard box on the shelf’								
	b.	L1 Danish:								
		*En*	*mand*	*stiller*	*en*	*kasse*	*på*	*en*	*reol*	
		A	man	puts.vertically	a	box	on	a	shelf	
	c.	L2 Danish:									‘A man (vertically) puts a box on the shelf’
		*En*	*mand*	*sætter*	*boksen*	*i*	*en*	*reol*		
		a	man	sets.vertically	box.the	in	a	shelf		
		‘A man (vertically) sets the box on a shelf’								

The other types of constructions had a differential relative frequency across the groups. PC #1 was mainly used for clothing scenes in the L1 Spanish group. PC #3 was employed by the L1 Danish group more frequently than by either the L2 Danish or the L1 Spanish groups. PC #4 was only used for encoding dressing events by the L1 and L2 Danish groups. PC #5 and PC#6 had a more restricted distribution: the former was predominantly found in the L1 Danish group and the latter, in the L2 Danish learner group.

Multinomial logistic regression models with ‘type of placement construction’ as dependent variable and ‘participant group’ as independent variable were used to examine whether there were significant differences between the three participant groups with respect to the type of construction used. Since PC #2 was the most common construction, it was used as the baseline category. Additionally, both the L1 and the L2 Danish groups were used as baselines. Given that the L1 Spanish NS group did not use PC #4 and PC #5, and only 3 respondents used PC #6, the statistical models were run only with PC #1, PC #2, and PC #3.

Tables 5a and 5b show the coefficients estimates, odds-ratio and *value of p*s of the multinomial regression models. In relation to the use of PC #1 vs. PC #2, there was no statistical difference between the baseline L1 Danish group and the L2 Danish group (*p*. = 0.919). In contrast, a statistical difference was found between the L1 Danish group and the L1 Spanish group in the use of PC #1 vs. PC #2 (*p*. = 0.001). This means that it was 2.712 times more likely for the L1 Spanish group than for the L1 Danish group to use PC #1 vs. PC #2. Additionally, a significant difference was found between the baseline L2 Danish group and the L1 Spanish group (*p*. = 0.001). Specifically, it was 2.617 times more likely for the L1 Spanish group than for the L2 Danish group to use PC #1 vs. PC #2.

**Table 5a:** Multinomial logistic regressions with PC #2 as baseline (L1-Danish baseline).

	Intercept	L1-spa	L2-dan	L1-spa	L2-dan
				Odds ratio	Odds ratio
PC #1	−2.419	0.998	0.036	2.712	1.036
Value of *p*	< 0.001	0.001	0.919	0.001	0.919
PC #3	−0.235	−1.071	−0.488	0.343	0.614
Value of *p*	0.031	< 0.001	0.003	< 0.001	0.003

dan = Danish; spa = Spanish.

**Table 5b:** Multinomial logistic regressions with PC #2 as baseline (L2-Danish baseline).

	Intercept	L1-dan	L1-spa	L1-dan	L1-spa
				Odds ratio	Odds ratio
PC #1	−2.383	−0.036	0.962	0.965	2.617
Value of *p*	*<* 0.001	0.919	0.001	0.919	0.001
PC #3	−0.723	0.488	−0.583	1.629	0.558
Value of *p*	< 0.001	0.003	0.003	0.003	0.003

dan = Danish; spa = Spanish.

In relation to the use of PC #3 vs. PC #2, a significant difference was found between the L1 Danish group and the L1 Spanish group (*p*. = 0.001). Specifically, it was 2.915 (=1/0.343) times more likely for the L1 Danish group than for the L1 Spanish group to use PC #3 vs. PC #2. Similarly, a significant difference was found between the L1 and L2 Danish groups in relation to the use of PC #3 vs. PC #2 (*p*. = 0.003). Specifically, it was 1.629 (=1/0.614) times more likely for the L1 Danish group than for the L2 Danish group to use PC #3 vs. PC #2. Finally, a significant difference was also found between the baseline L2 Danish group and the L1 Spanish group in the use of PC #3 vs. PC #2 (*p*. = 0.003). This means that it was 1.792 (=1/0.558) times more likely for the L1 Spanish group than for the L2 Danish group to use PC #2 vs. PC #3.

In order to obtain comparisons between PC #1 and PC #3, another multinomial logistic regression with PC #3 as the baseline was run. As in the previous models, the L1 and the L2 Danish groups were used as baselines. Tables 6a and 6b below show the coefficients estimates, odds-ratio and *value of p*s of these multinomial regression models. Note that the odd-ratios of PC #2 in Tables 6a and 6b are now the inverse of the odd-ratios of PC #3 in Tables 5a and 5b and will not be commented any further.

**Table 6a:** Multinomial logistic regressions with PC #3 as baseline (L1-Danish baseline).

	Intercept	L1-spa	L2-dan	L1-spa	L2-dan
				Odds ratio	Odds ratio
PC #1	−2.184	2.069	0.524	7.916	1.688
Value of *p*	< 0.001	< 0.001	0.143	< 0.001	0.143
PC #2	0.235	1.071	0.488	2.918	1.629
Value of *p*	0.031	< 0.001	0.003	< 0.001	0.003

dan = Danish; spa = Spanish.

**Table 6b:** Multinomial logistic regressions with PC #3 as baseline (L2-Danish baseline).

	Intercept	L1-dan	L1-spa	L1-dan	L1-spa
				Odds ratio	Odds ratio
PC #1	−1.661	−0.523	1.545	0.593	4.689
Value of *p*	< 0.001	0.144	< 0.001	0.144	< 0.001
PC #2	0.723	−0.488	0.583	0.614	1.792
Value of *p*	< 0.001	0.003	0.003	0.003	0.003

dan = Danish; spa = Spanish.

When including PC #3 as baseline, there was again a significant difference between the baseline L1 Danish group and the L1 Spanish group in the use of PC #1 (*p*. = 0.001). Specifically, it was 7.916 more likely for the L1 Spanish group than the L1 Danish group to use PC #1 vs. PC #3. Additionally, a significant difference was found between the baseline L2 Danish group and the L1 Spanish group in the use of PC #1 vs. PC #3 (*p*. = 0.001). This means that it was 4.689 times more likely for the Spanish L1 than for the L2 Danish learner group to use PC #1 vs. PC #3. In contrast, no significant difference was found again between the L2 Danish and the L1 Danish groups in the use of PC #1 vs. PC #3 (*p*. = 0.143).

Finally, a Log-ratio test was conducted in order to verify whether including the ‘participant group’ variable in the model improved the fitness of the data. In other words, the null hypothesis was that the coefficient of the ‘participant group’ variable was zero vs. the alternative hypothesis of non-zero coefficient of the ‘participant group’ variable. The empirical value of the test was $\chi^{2}$ =64.146 and the *value of p* < 0.001. This result is consistent with the claim that the ‘type of construction’ variable is significantly related to the ‘participant group’ variable. The post-hoc analysis with Bonferroni correction showed significant differences in the pairwise comparisons (i.e., L1 Spanish vs. L1 Danish; L1 Spanish vs. L2 Danish; L1 Danish vs. L2 Danish) with *p* < 0.001.

### Frequency of spatial particles used by the three participant groups

Given that PC #3 was a predominant type of construction in the data, we examined the use of the spatial particles that are characteristic of this construction. Table 7 shows the spatial particle types and tokens used by each participant group.

**Table 7:** Use of spatial particle types and tokens by the three participant groups.

L1 Spanish		L1 Danish		L2 Danish	
*Types*	Tokens	*Types*	Tokens	*Types*	Tokens
*encima* ‘above’	37	*ned* ‘down’	113	*ind* ‘in-directional’	99
*dentro* ‘inside’	18	*på* ‘on’	38	*på* ‘on’	21
		*op* ‘up’	25	*over* ‘over, across’	16
		*ind* ‘in-directional’	14	*inde* ‘in-locational’	11
		*oven* ‘above’	9	*ned* ‘down’	10
		*ud* ‘out’	6	*op* ‘up’	9
		*over* ‘over, across’	4	*oven* ‘above’	9
		*tilbage* ‘back’	3	*ud* ‘out’	7
		*bag* ‘behind’	1	*af* ‘off’	2
		*hen* ‘along’	1	*om* ‘about, around’	1
		*inde*‘in-locational’	1		
		*nede* ‘down-static’	1		
*2*	55	*12*	216	*10*	185

As shown in Table 7, L1 Spanish NSs used a total of 55 tokens and only 2 types of particles (type-token ratio = 0.036). The particles used were the spatial nominal adverbs *dentro* ‘inside’ and *encima* ‘on top of’. The use of these adverbs in PC #3 is not compulsory; they do not add any further spatial information beyond the canonical placement of these objects. Cups are usually on tables rather than vice versa or underneath them. The use of these adverbs seems to reinforce this spatial configuration, as in *Pone la taza encima de la mesa* ‘(he/she) puts the cup on top of the table’. In contrast, L1 Danish NS used a wider range of spatial particles, whichwere used more frequently; namely, 216 tokens and 12 types (type-token ratio = 0.056). The most frequent particle was *ned* ‘down’, followed by *på* ‘on’, and *op* ‘up’. The higher frequency of these spatial particles in the Danish NS data is natural considering that one of the most frequently used BPCs in Danish included such particles in its formal structure, although the type, diversity and frequency of the particles found in the data are also dependent on the types of scenario presented in the elicitation material.

Finally, the L2 Danish learner group used a total of 185 tokens and 10 types (type-token ratio = 0.054). Even though the type-token ratio in the L1 and L2 Danish data was similar, their use of spatial particles differed in two ways. First, in the frequency with which *specific* particles were used. In contrast to the L1 Danish NS data, the most frequently used spatial particle employed by the learner group was *ind* ‘in-directional’, followed by *på* ‘on’, followed by *over* ‘over, across’, *inde* ‘in-locational’, *ned* ‘down’, *op* ‘up’, *oven* ‘above’, *ud* ‘out’, *af* ‘off’, and *om* ‘about, around’. Second, the L2 learners used two spatial particles that were not found in the Danish NS data, namely *af* ‘off’ and *om* ‘about, around’. The former was predominantly found in PC #6. This construction was used to describe the scene drop book accidentally on floor [009]. The latter turned up in PC #2 used in descriptions for scene put saucer on top of cup [031]. Examples (3) and (4) illustrate both particles, respectively.

**Table:** 

(3)	*Mens*	*en*	*mand*	*bærer*	*nogen*	*bøger,*	*en*	*af*	*dem*	*falder*	*af*
	while	a	man	carries	some	books,	one	of	them	falls	off
	‘While a man carries some books, one of them falls off’										
(4)	*Der*	*er*	*en*	*person*	*som*	*tager*	*en…*	*sætter*		*en*	
	there	is	a	person	who	takes	one…	sets.vertically		a	
	*tallerken,*		*lille*	*tallerken*		*om*	*på*	*en*	*stor,*	*en*	*stor*
	plate,		little	plate		about	on	a	big,	a	big
	*kop*										
	cup										
	‘There is a person who takes one… sets a plate, little plate about on a big, a big cup’										

## Discussion

The present study addressed two research questions. The first question asked what types of placement constructions were used by a group of L1-Spanish learners of L2 Danish and two groups of NSs (Spanish and Danish) when describing placement events. The second question addressed the issue of frequency usage for the different types of placement constructions in the three participant groups.

### Placement constructions used by the three participant groups

In relation to the first research question, results showed that the three participant groups employed six different types of constructions. Five constructions (PC #1, PC #2, PC #3, PC #4, and PC #5) were caused-motion, and one construction, PC #6, provided a stative description divided into two clauses as illustrated in (5). The first clause describes the location of the Figure before the caused-motion event takes place whereas the second clause describes where and how the Figure ended up after the caused-motion event takes place (see also Cadierno, 2010; Hijazo-Gascón et al., 2016).

**Table:** 

(5)	*En*	*mand*	*bærer*	*nogle*	*bøger*	*men*	*en*	*af*	*dem*	*falder*
	a	man	carries	some	books	but	one	of	them	falls
	‘A man carries some books and one of them falls’									

The first five types of caused-motion constructions varied with respect to the number of elements and thus, their syntactic and semantic complexity; i.e., the number of syntactic and semantic units. For example, PC #1 does not incorporate a NP/PRO encoding the Ground while the others do express the location to which the Figure is moved to. Likewise, PC #3 can be considered more complex than PC #2 as it encodes both a PART encoding the Path of motion (i.e., *op* ‘up’) and a PP encoding the Ground (*på reolen* ‘on the shelf’). In contrast, PC #2 only encodes the latter. In turn, PC #5 seems to be more complex than PC #3 as it involves the use of more than one PART and one PP. This means that PC #5 offers information about more than one Path of motion as well as more than one Ground as illustrated in (6). In this example, the spatial particles, *ud* ‘out’ and *ned* ‘down’, describe Paths of motion and the prepositional phrases, *af koppen* ‘of the cup’ and *på jorden* ‘on the ground’, two Grounds.

**Table:** 

(6)	*Hun*	*hælder*	*vand*	*ud*	*af*	*koppen*		*ned*	*på*	*jorden*
	she	pours	water	out	of	cup.the		down	on	ground.the
	‘She pours water out of the cup down onto the ground’									

However, as shown in Table 2, the different types of placement constructions were not used with the same frequency by the participant groups. PC #2 was the most widely used type of construction followed by PC #3, PC #6, PC #4, PC #1, and PC #5. This pattern may be partly explained by the nature of the video scenes that were shown to the participants. For example, PC #4 and PC #1 were primarily used to describe dressing scenes in Danish and Spanish respectively, but in the stimuli, only three out of the 31 short video clips depicted dressing events (namely, put hat on head [025], put boot on foot [026], and put on coat [033]). The low frequency of PC #5 and PC #6 can also be expected since they are not the default type of construction in caused-motion events in Danish. PC #5 involves the use of longer and more complex structure with the corresponding higher degree of cognitive effort on the part of the participants to process it, and PC #6 is not a caused-motion construction itself.

### Frequency in the use of different types of placement constructions by the three participant groups

The second research question asked about possible differences in the types of placement constructions employed by the Spanish and Danish NSs groups and the learner group. For the sake of clarity of exposition, we first discuss the differences observed between the two NS groups and secondly, the differences between the learner group vis-a-vis the two NS groups.

#### Differences between the two NS groups

Results showed that the Danish and Spanish NS groups differed with respect to the type of construction that was more frequently used. Specifically, and as shown in Table 3 and Figure 1, Danish NSs used two predominant types of constructions, i.e., PC #2 and PC #3, whereas the Spanish NS only used PC #2 as predominantly. As argued in Hijazo-Gascón et al. (2016), Danish thus has two Basic Placement Constructions whereas Spanish only has one. In fact, the results of the multinomial logistic regressions showed significant differences between the two NS groups in relation to the use of these two constructions, with the Danish NSs using PC #3 to a significantly greater extent (relative to PC #2) than the Spanish NSs (see Table 5a), and with the Spanish NSs using PC #2 to a greater extent (relative to PC #3) than the Danish NSs (see Table 6a). Given the higher frequency of use of PC #3 by the L1 Danish group, the frequency of particles used by L1 Danish speakers is also higher than the one used by Spanish L1 speakers. Specifically, the Danish speakers used a total of 216 tokens and 12 types (type-token ratio: 0,056) whereas the Spanish speakers used a total of 55 tokens and only 2 types (type-token ratio: 0,036). This finding is in line with Talmy’s (1991, 2000) typological framework on the expression of motion events. Danish, as a satellite-framed language, typically encodes the Path of motion outside the main verb, in a satellite such as *op* ‘up’, *ned* ‘down’, *ind* ‘in-directional’, or *ud* ‘out’. In Spanish, on the other hand, the use of spatial adverbs is much more restricted and less frequent (Ibarretxe-Antuñano, 2012; Hijazo-Gascón et al., 2016; Hijazo-Gascón, 2021).

The two NS groups also differed in relation to the frequency of use of PC #4 and PC #5. PC #4 is not possible in Spanish and this is why it did not turn up in the L1 Spanish data. PC #5, on the other hand, may be possible in Spanish. However, due to its complex form –it encodes more than one Path and/or Ground– is quite unlikely to appear in Spanish. Verb-framed languages such as Spanish do not tend to express Path of motion in external satellites and thus, motion event descriptions by Spanish NSs tend to be less detailed in relation to the expression of the trajectories involved in motion events (Slobin, 1996, 2004). Finally, the two NS groups also differed in relation to their use of PC #1, which, as shown in Tables 5a and 6a, was used to a significantly higher degree by the Spanish NSs than by the Danish NSs (both in relation to PC #2 and PC #3). As mentioned in “Relative frequency of placement construction type per participant group”, PC #1 is typically used for dressing events in L1 Spanish. This construction includes the use of the clitic *se* together with *poner* ‘put’. The result, *ponerse*, triggers a reflexive meaning ‘put to oneself’. PC #1 was also used in other scenes where the use of the clitic *se* did not correspond to a reflexive reading, but marked the degree of intentionality of the event. PC #1 is used to trigger the unintentional or accidental character of events in scenes such as drop book accidentally on floor [009] in contrast with intentional scenes such as drop book deliberately on floor [008], and toss book on floor [010] (Ariño-Bizarro and Ibarretxe-Antuñano, 2020, under review; Ibarretxe-Antuñano et al., 2016). Furthermore, in the Spanish NS data, PC #1 is generally used alone without any further elaboration of the Ground information (e.g., *Se le cayó el libro* ‘The book fell on him’). In line with previous findings (Slobin, 1996, 2004; Cadierno, 2004; Hijazo-Gascón, 2021), this tendency to mention fewer Grounds in verb-framed languages such as Spanish also arise in these data.

#### Differences between the learner group and the two NS groups

Again, in relation to the second research question, results showed differences between the L2 Danish learner group and their corresponding L1 Danish and L1 Spanish NS groups with respect to the type of constructions that were used to describe placement events. Overall, the L2 Danish learners exhibited a similar pattern to the one shown by the target L1 Danish group, with two types of constructions being used more frequently; namely, PC #2 and PC #3. However, when compared with the Danish NS, the L2 Danish learners exhibited a slightly more frequent use of PC #2 (i.e., the only BPC in L1 Spanish) and a less frequent use of PC #3, i.e., a type of construction that is rather infrequent in their L1 Spanish.

In fact, results of the multinomial logistic regressions showed significant differences between the L2 Danish learners and the L1 Danish NS group in relation to the use of these two constructions. The L1 Danish NSs used PC #3 (vs. PC #2) to a significantly greater extent than the L2 Danish learners (see Table 5b), and the L2 Danish learner group used PC #2 (vs. PC #3) to a greater extent than the Danish NSs (see Table 6b). Interestingly, the L2 learner group’s performance also differed from the Spanish NS group. As shown in Table 5b, the learner group employed PC #3 (vs. PC #2) to a significantly higher degree than the L1 Spanish NS.

These findings suggest the existence of an in-between performance, i.e., the creation of a linguistic conceptualization pattern (a specific pattern of form-meaning pairings) on the part of the learners that is different from the respective L1 and L2 monolingual patterns. This is a hybrid type of behavior also documented in previous research (e.g., Brown and Gullberg, 2012; Treffers-Daller and Tidball, 2016; Madlener-Charpentier, 2022; see “L2 acquisition”). These results thus provide empirical support for Grosjean’s (1982) and Cook’s (1992) notion of bilinguals’ multicompetence not being equivalent to those of two monolinguals. Furthermore, the less frequent use of PC #3 on the part of the learners can be explained by two facts: (i) they are not used to paying so much attention to directionality in their L1 Spanish as evidenced by the low frequency of PC #3 in the Spanish NS data; and (ii) PC #3 is a more semantically and grammaticality complex construction than PC #2. Given the descriptive nature of the present study, it is difficult to tease apart the role of these factors (i.e., frequency and complexity) because they could in isolation or in combination explain the patterns found in the L2 data. The existence of an in-between performance on the part of the learner group is also reflected in their use of spatial particles in PC #3. L2 learners used a greater number of spatial particles (tokens and types) than L1 Spanish NS but a lower number than L1 Danish NS. In addition, when zooming in on the specific spatial particles used, differences also arise: spatial particles were more frequently used by the L2 and L1 Danish group than by L1 Spanish NS. For the L1 Danish group, the most frequently used spatial particle was *ned* ‘down’, whereas, for the L2 Danish group, it was *ind* ‘in-directional’.

A qualitative look at the data also revealed interesting differences in the way in which the two Danish groups employed these two spatial particles. For example, when describing the scene drop apple into bag [012] and put stone into pocket [016], L1 Danish speakers preferred utterances such as those in (7). L2 learners, on the other hand, did not follow this pattern but preferred sentences such as those in (8) for the same scenes.

**Table:** 

(7)	L1 Danish										
	a.	*En*	*mand*	*putter*	*et*	*æble*	*ned*	*i*	*en*	*pose*	
		a	man	puts	an	apple	down	in	a	bag	
		‘A man puts an apple down in a bag’									
	b.	*Damen*	*putter/lægger*	*noget*		*ned*	*i*	*sin*	*lomme*		
		woman.the	puts/puts.horizontally	something		down	in	her	pockert		
		‘The woman puts/lays something down in her pocket’									
(8)	L2 Danish										
	a.	*En*	*mand*	*putter*	*et*	*æble*	*ind*	*i*	*en*	*pose*	
		a	man	puts	an	apple	in-directional	in	a	bag	
		‘A man puts an apple into a bag’									
	b.	*Nogen*	*putter*	*en*	sten	*ind*	*i*	*lomme*			
		somebody	puts	a	stone	in-directional	in	pocket			
		‘Somebody puts a stone into the pocket’									

These differences were not isolated but also arise in other scenes such as put stone into pot of water [019] and put candle into candle stand [014]. In all these cases, L2 learners seem to be transferring the linguistic conceptualization of their L1 Spanish. In L1 Spanish, these scenes are usually encoded by means of specific placement verbs such as *meter* ‘put.in’ and *introducir* ‘introduce’ which mark the container information in their semantics as illustrated in (9).

**Table:** 

(9)	a.	*Mete/introduce*	*la*	*manzana*	*dentro*	*de/en*	*la*	*bolsa*
		puts.in/introduces	the	apple	inside	of/loc	the	bag
		‘He puts the apple in the bag’							
	b.	*Mete*	*la*	*piedra*	*en*	*la*	*olla*	
		puts.in	the	stone	loc	the	pot	
		‘She puts the stone in the pot’							

That is, Spanish NSs conceptualize these placement events as involving a containment type of relationship between the Figure and the Ground. In contrast, Danish NS also pay attention to the downward verticality of the placement movement. What Spanish learners of L2 Danish do is different: they follow the constructional tendency in L1 Danish, that is, the use of a spatial particle. However, the particle they choose, *ind* ‘in-directional’, reflects a different pattern. The use of *ind* ‘in-directional’ indicates that Danish L2 learners are still guided by their L1 Spanish pattern; that is, the containment relationship.

The use of non-caused-motion PC #6 is also noticeable in the case of L2 Danish learners. As illustrated in example (5) above, learners seem to divide the caused-motion event into two or more sub-events by means of different clauses. Whereas the first clause(s) tells the location of the Figure before the caused-motion event takes place, the second clause describes where and how the Figure ended up after the caused-motion event occurs. Interestingly, the same pattern of use was found in the L2 expression of spontaneous motion events involving boundary-crossing (Cadierno, 2010).

In sum, crucial significant differences were found between the Spanish and the Danish NS groups when describing the same placement events. With respect to the L2 learners, findings suggest the creation of a linguistic conceptualization pattern that is different from the respective L1 and L2 monolingual patterns and the influence of their L1 conceptualization patterns.

## Conclusion

The present study adopted a constructional perspective to the study of placement events by L1 and L2 speakers. This is an innovative take on the acquisition of this type of events, as previous research has mainly focused on the acquisition of placement verbs. The fact that the target language is satellite-framed makes this constructional approach even more justified, as some of the most important semantic elements are encoded out of the main verb of the event. The study also contributes to the need of further research in L2s other than English and with different language combinations (Hijazo-Gascón and Llopis-García, 2019).

The results of the study showed important cross-linguistic differences in how native speakers of Danish and Spanish talk about placement. First, differences arise with respect to the choice and number of Basic Placement Constructions in these languages. Danish NS used two predominant types of placement constructions. These are: PC #2 [NP/PRO + V + NP/PRO (DO) + PP (WHERE) (+ PP/gerund (INST))] and PC #3 [NP/PRO + V + NP/PRO (DO) + PART (PATH) + PP (WHERE) (+ PP/Gerund (INST))]. Spanish NS, on the other hand, only used one predominant construction, namely, PC #2. In fact, significant differences were found between the two NS groups in relation to the use of these two constructions. Second, significant differences were found between the two NS groups with respect to the use of PC #1 [NP/PRO + V + NP/PRO (DO) (+ PP/Gerund (INST))] when comparing the frequency of this construction in relation to PC #2 and PC #3. In both cases, the Spanish NS group made more frequent use of this construction than Danish NSs. Third, differences between the two NS groups were found in relation to the use of spatial particles. L1 Danish NS used a wider range of spatial particles (both tokens and types) than L1 Spanish NS.

The cross-linguistic differences found in the way Danish and Spanish talk about placement lead to difficulties in L2 acquisition of Danish by Spanish learners. The learner group’s performance in relation to the use of PC #3 (vs. PC #2) was significantly different from that of both the L1 Danish and L1 Spanish NS groups. Additionally, the frequency with which the L2 learners used spatial particles (tokens and types) also evidenced an in-between performance with respect to the frequencies of the two NS groups. These findings have been interpreted as providing empirical evidence for Grosjean’s (1982) and Cook’s (1992) notion of bilinguals’ multicompetence not being equivalent to those of two monolinguals. Furthermore, results from the L2 learners also showed the influence of their L1 linguistic conceptualization patterns when describing placement events in L2 Danish. This was evidenced in the less frequent use of PC #3 and the inappropriate use of spatial particles. Therefore, learners need to learn that PC #3 (i.e., a construction that includes explicit spatial particles) is a frequent type of construction in L2 Danish to describe placement events, and they also need to learn the L2 appropriate conceptualization of the spatial particles in order to develop a better command of target language meanings. Finally, the results showed that learners resorted to non-caused-motion constructions as a communicative strategy. In conclusion, it seems that the difficulties in re-thinking-for-speaking (Cadierno, 2008) are present in Spanish learners of L2 Danish, as they were in Danish learners of L2 Spanish (Hijazo-Gascón et al., 2016).

One issue that has not been analyzed in the present study, and which merits further research, is the extent to which the types of constructions that are employed by the participant groups are correlated with the types of verbs that appear in the constructions. In other words, do verbs that show specific conflational patterns (e.g., Path and Motion vs. Manner of motion) tend to occur in different types of constructions? Such an analysis would help elucidate whether differences in the types of constructions employed in Danish and Spanish are intrinsically related to differences in the prototypical conflational verb patterns in each language. Additionally, the present study has not examined the types of constructions that are used in relation to specific placement scenes. Although some observations have been made in relation to the use of PC #4 as specific to Danish description of dressing events, future research could examine this phenomenon in a more systematic way.^3^

Some pedagogical implications can be drawn from these results. There is a clear need to raise learners’ awareness towards the semantic domain of caused-motion and the linguistic conceptualization of everyday events. Foreign language teachers could use Contrastive Linguistics in class, pointing out at similarities and differences between the L1 and L2, with special attention to problematic cases (e.g., encoding of the position of the object in Danish placement verbs, use of spatial particles), use of video-clips, etc. The Focus on Form approach with insights from Language Typology, e.g., Applied Language Typology (Filipović, 2017), could be an excellent perspective to bring these language contrasts to the second language instruction (Cadierno, 2008; Hijazo-Gascón et al., 2016), also in relation to Vygotsky’s Sociocultural Theory (Aguiló-Mora and Negueruela, 2015). Another teaching perspective is that of Pedagogical Translation (González Davies, 2004; Leonardi, 2010) through which learners’ awareness of the typological contrasts between the L1 and the L2 could be raised (Hijazo-Gascón, 2021). This technique could also help to develop mediation skills, a key component of plurilingual competences, defined as the language activities that make communication possible between persons who are unable to communicate with each other (Council of Europe, 2001, 2018). Further research with pedagogical interventions would shed more light on how these contrasts can be better acquired. Similarly, future research on placement events in different language pairs and with learners with different levels of proficiency will also contribute to gain a better understanding on how language typological contrasts challenge the acquisition of a second language.

## Data availability statement

The datasets presented in this article are not readily available because Data cannot be accessed without revealing the identity of the participants. Requests to access the datasets should be directed to cadierno@sdu.dk.

## Ethics statement

Ethical review and approval was not required for the study on human participants in accordance with the local legislation and institutional requirements. The patients/participants provided their written informed consent to participate in this study.

## Author contributions

All authors listed have made a substantial, direct, and intellectual contribution to the work and approved it for publication.

## Funding

Funds for the publication of this article were provided by the AEI project CONESSO FFI2017-82460-P. Research reported in this paper has been funded by the Spanish Ministry of Science and Innovation (CONESSO FFI2017-82460-P; MOTIV PID2021-123302NB-I00), the Spanish Ministry of Universities and the European Union-Next GenerationEU (María Zambrano Program MZ-240621), the Government of Aragon (Psylex H11-17R; MultiMetAR LMP143_21) and the Iberus Campus (ICON action group).

## Conflict of interest

The authors declare that the research was conducted in the absence of any commercial or financial relationships that could be construed as a potential conflict of interest.

## Publisher’s note

All claims expressed in this article are solely those of the authors and do not necessarily represent those of their affiliated organizations, or those of the publisher, the editors and the reviewers. Any product that may be evaluated in this article, or claim that may be made by its manufacturer, is not guaranteed or endorsed by the publisher.

## Figures and Tables

**Figure:**
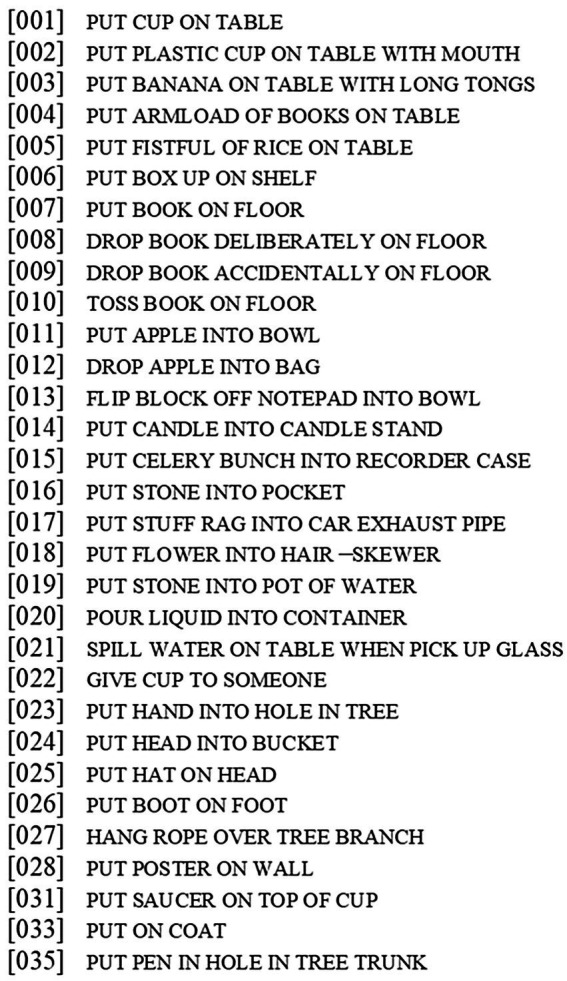
Video clips used in this study.

## Footnotes

^1^This section reviews studies with second language learners, focused on the second language acquisition process but see Berthele (2012) and Alferink and Gullberg (2014) for early bilinguals; van Bergen and Flecken (2017) for predictive processing; and Koster and Cadierno (2019) for language and memory.

^2^Debates about their morpho-syntactic relations and dependencies as well as about their formal integration within the clauses and sentences they appear in are still open, complex, and inconclusive in the literature (see Stolova, 2015 for a review). The focus in this study relies on the semantic role that these "particles" play in the overall construction of placement events and how they affect the semantic categorization of these events in L2 learners. Each language has its own linguistic means to encode placement events including problematic elements such as those mentioned above. The scope of the cover term "spatial particle" is just restricted to practical reasons (comparison between construction types in different languages). A deeper debate on the formal characterization of these "spatial particles" is, therefore, beyond the scope of this paper.

^3^We thank an anonymous reviewer for these insightful comments.

## Appendix 1

Description of video clips used in this study. Codes and labels are shown in small caps by convention (see Bowerman et al., 2004).
